# Systems Network Pharmacology-Based Prediction and Analysis of Potential Targets and Pharmacological Mechanism of *Actinidia chinensis* Planch. Root Extract for Application in Hepatocellular Carcinoma

**DOI:** 10.1155/2022/2116006

**Published:** 2022-09-20

**Authors:** Yue Hu, Liang Yang, Yunfei Lu, Yong Wang, Jianshuai Jiang, Yahui Liu, Qing Cao

**Affiliations:** ^1^Department of Hepatobiliary and Pancreatic Surgery, Ningbo First Hospital, Ningbo, Zhejiang, China; ^2^Medical Record Statistics Department, Ningbo First Hospital, Ningbo, Zhejiang, China; ^3^Department of Scientific Research, Hwa Mei Hospital, University of Chinese Academy of Sciences, Ningbo, Zhejiang, China

## Abstract

**Purpose:**

Traditional Chinese medicine (TCM) sometimes plays a crucial role in advanced cancer treatment. Despite the significant therapeutic efficacy in hepatocellular carcinoma (HCC) that *Actinidia chinensis* Planch root extract (acRoots) has proven, its complex composition and underlying mechanism have not been fully elucidated. Therefore, this study analyzed the multiple chemical compounds in acRoots and their targets via network pharmacology and bioinformatics analysis, with the overarching goal of revealing the potential mechanisms of the anti-HCC effect.

**Methods:**

The main ingredients contained in acRoots were initially screened from the traditional Chinese Medicine Systems Pharmacology Database and Analysis Platform (TCMSP), and the candidate bioactive ingredient targets were identified using DrugBank and the UniProt public databases. Second, the biological processes of the targets of active molecules filtered from the ingredients of acRoots were evaluated using gene ontology (GO) enrichment and Kyoto Encyclopedia of Genes and Genomes (KEGG) pathway analyses. Third, weighted gene coexpression network analysis (WGCNA) was performed to identify gene coexpression modules associated with HCC. The hub genes of acRoots in HCC were defined via contrasting the above module eigengenes with candidate target genes of acRoots. Furthermore, the target-pathway network was analyzed to explore the mechanism for anti-HCC effect of hub genes. Kaplan–Meier plotter database analysis was performed to validate the hub genes of acRoots correlation with prognostic values in HCC. In order to verify the results of the network pharmacological analysis, we performed a molecular docking approach on the active ingredients and key targets using the Discovery Studio software. The viability of SMMC-7721 and HL-7702 cells was determined by Cell counting kit-8 (CCK-8) after being treated with different concentrations of (+)-catechin (0, 50, 100, 150, 200, and 250 g/ml) for 24, 48, and 72 hours, respectively. Finally, qRT-PCR and Western blot involving human hepatocarcinoma cells were utilized to verify the impact of (+)-catechin on the hub genes associated with prognosis.

**Results:**

6 out of 26 active ingredients extracted from TCMSP were deemed as the core ingredients of acRoots. 175 bioactive-ingredient targets of acRoots were obtained and a bioactive-ingredient targets network was established correspondingly. The biological processes (BP) of target genes mainly involved processes, such as toxic substance and wounding. The results of KEGG pathways indicated that the target genes were mainly enriched in pathways in cancer, AGE-RAGE signaling pathway in diabetic complications, IL-17 signaling pathway, and other pathways. Also, the two hub genes (i.e., ESR1 and CAT) were closely associated with the prognosis of HCC patients. As a consequence, we predicated a series of signaling pathways, including estrogen signaling pathway and longevity regulation pathway, through which acRoots could facilitate the treatment for HCC. The molecular docking experiment ascertained that ESR1 and CAT had an effective binding force with (+)-catechin, one of the core ingredients of acRoots. Furthermore, (+)-catechin inhibited SMMC-7721 cell growth in a dose-dependent manner and a time-dependent manner. Finally, we suggest that the expression level of ESR1 and CAT is positively related to the (+)-catechin concentrations in in-vitro experiments.

**Conclusion:**

The bioactive ingredients of acRoots, including quercetin, (+)-catechin, beta-sitosterol, and aloe-emodin, have synergistic interactions in reinforcing the anticancer effect in HCC. Evidently, acRoots took effect by regulating multitargets and multipathways through its active ingredients. Further, (+)-catechin, the possible paramount anti-HCC active ingredient in acRoots, helped improve the prognosis of HCC patients by increasing the expression of ESR1 and CAT. Additionally, the findings yielded provide a conceptual guidance for the clinical treatment of HCC and the methods adopted are potentially applicable in the future comprehensive analysis of the underlying mechanisms of TCMs.

## 1. Introduction

Hepatocellular carcinoma (HCC) remains one of the most common cancers and primary causes of cancer-related death worldwide, due to its high recurrence rate and poor prognosis [1]. It is noteworthy that China retains a relatively high prevalence rate of liver cancer, with approximately half of the global mortality caused by HCC [2]. At present, HCC is mainly treated by surgery combined with interventional therapy, chemotherapy, targeted therapy, and immunotherapy [3]. Traditional Chinese medicine (TCM) has been employed to treat diseases for centuries in China and is also extensively used in other Asian nations. Studies ascertain that TCM is a pivotal therapeutic approach in advanced cancer treatment [4, 5]. Given that TCM provides a rich source of compounds for new drug discovery and has been gradually recognized as an effective complementary medicine for cancer treatment, it has a huge potential for HCC treatment [5].

The roots of *Actinidia chinensis* Planch, regardless of its historic role in TCM, demonstrated strong inhibition against tumor growth in various types of human cancer cells, including HCC cells. The main antitumor active compounds extracted from *A*. *chinensis* Planch. roots are triterpenoids, polysaccharides, and phenolic compounds, which have proven effective in mitigating HCC [6]. Nonetheless, the complexity of what acRoots consists of and the underlying mechanisms of how it takes effect have been still underexplored. Therefore, this calls for studies exploring how to accurately extract active ingredients in acRoots, minimize toxic side effects, and enhance anticancer effects.

The network pharmacology method and bioinformatics analysis provide a new method for analyzing the multiple chemical compounds and their targets, with the overarching goal of unravelling the potential underlying mechanisms [7, 8]. In recent years, network pharmacology or bioinformatics analysis has been widely adopted to investigate cancer treatments and identify multiple interactive networks of “Disease-Gene-Target-Medicine” [9, 10]. Therefore, it is expected that these research strategies will effectively analyze the anticancer active ingredients of TCM and elucidate its underlying anticancer mechanisms.

Firstly, the whole process of the present study is fivefold screened out the main ingredients in *A*. *chinensis* Planch roots and identified the candidate bioactive ingredient targets from several online databases. Secondly, weighted gene coexpression network analysis (WGCNA) was performed to identify gene coexpression modules associated with HCC. To define hub genes, further analyses were conducted via contrasting the above module eigengenes with candidate target genes of acRoots. Subsequently, gene ontology (GO) enrichment and Kyoto Encyclopedia of Genes and Genomes (KEGG) pathway analysis were performed using the Metascape functional annotation tool. Furthermore, Kaplan–Meier plotter database analysis was employed to validate the correlation between hub genes of acRoots and prognostic values in HCC. Collectively, these results will provide insights for clinical application of acRoots in HCC treatment.

## 2. Material and Methods

### 2.1. Screening of Chemical Ingredients in the Roots of *A. chinesis*

All the research data analyzed here were obtained from domestic and foreign research databases. The main ingredients contained in the roots of *A. chinensis* Planch were screened from the traditional Chinese Medicine Systems Pharmacology Database and Analysis Platform (TCMSP), which is a specialised platform for Chinese herbal medicines based on systems pharmacology [11]. The ADME parameters used in this study include predicted oral bioavailability (OB) and drug-likeness (DL). Specifically, OB is one of the most important pharmacokinetic parameters used in screening of orally administered drugs, which indicates the speed and degree to which a given active drug ingredient may be absorbed to human circulation. On the other hand, DL refers to the similarity between ingredients and known drugs, which is a key factor in evaluating drug efficacy. In this study, the screening conditions were set to satisfy both OB ≥ 30% and DL ≥ 0.18, in accordance with a previously published study [7].

### 2.2. Fishing for Candidate Targets of the Ingredients

In organisms, the active ingredients of drugs usually interact with targets to exert their biological functions. In this study, fishing of protein targets associated with active ingredients of *A. chinensis* Planch. roots was performed by using DrugBank public database [12]. Subsequently, all corresponding targets were converted into gene names by searching the UniProt human database [11]. All of the obtained target genes were then exported to Excel, followed by the removal of duplicates. The final candidate bioactive ingredient targets were identified after the abovementioned screening.

### 2.3. Network Construction and Enrichment Analysis of Bioactive Ingredient Targets

To further uncover the synergistic roles of multitargets and multifunctions of active pharmaceutical ingredients in *A. chinensis* Planch roots, a compound-target (C-T) network of the drug was constructed using Cytoscape [13]. Next, gene ontology (GO) enrichment and Kyoto Encyclopedia of Genes and Genomes (KEGG) pathway analyses were performed using the Metascape functional annotation tool to explore the main biological processes of the targets of active molecules that we filtered from the ingredients of *A. chinensis* Planch roots. Notably, GO and KEGG terms are sorted by *P*-values ranging from small to large [14]. The lower the *P*-values were, the more significant the terms were regarded. Only GO and KEGG terms with adjusted *P*-values of  ≤0.05 were subjected to further pharmacologic analysis.

### 2.4. Collection and Preparation of Genetic and Clinical Data

To explore the clinical significance of the predicted targets of *A. chinensis* Planch root ingredients, the clinical data was retrieved from the Gene Expression Omnibus (GEO) database [15]. The GSE10141 dataset included the mRNA sequencing expression profile of 80 hepatocellular carcinoma samples and 82 nontumor liver tissues samples and the clinical data of 80 hepatocellular carcinoma patients. Additionally, the gene expression dataset was obtained using Affymetrix GPL5747 platform.

The “edgeR23” R package was used to screen differentially expressed genes (DEGs) between normal and cancer samples, with an adjusted *P* < 0.05 and a fold control (log2|FC|) >1 as the cutoffs. Microarray data were normalized by rma from the “affy” package in R (R 3.2.0), followed by log10‐transformation. Probes were then mapped onto distinct genes on the basis of the current annotation files. Finally, the mean level of gene expression was calculated.

### 2.5. Construction of Coexpression Network Module Analysis

After preprocessing the GSE10141 data, a weighted gene coexpression network was built from the expression values of 6100 genes using the weighted gene coexpression network analysis (WGCNA) method in R package [16]. According to the correlation-strength based group genes, a hierarchical cluster tree was constructed subsequently. It should be noted that outlier samples were excluded to guarantee the most stable clustering. In addition, the correlations between modules and clinical features were identified by Pearson's correlation tests to select modules of interest that were significantly associated with clinical information. The clustering heatmap was then created with the “pheatmap” R package to depict the strength of interactions.

### 2.6. Identification of Hub Genes and Construction of Protein-Protein Interaction (PPI) Network

A Wayne diagram was used to identify the common genes by matching candidate targets of the active ingredients of *A. chinensis* Planch roots and the genes associated with the key modules. These common genes were considered as core targets of anticancer active ingredients in acRoots. The synergistic interaction between all core genes of anticancer active ingredients was evaluated by constructing protein-protein interactions (PPI) in the online STRING database [17]. The resulting data of the PPI network were then imported into Cytoscape software to visualize the relationships. It is worth mentioning that hub genes were genes with a large degree of the nodes and played a key role in the PPI network and were identified according to the degree of correlation nodes (genes with a node degree of ≥10 were selected as hub genes in the PPI network). Functional gene annotation and gene enrichment analysis were performed using Metascape online database. The signal pathway network of active Chinese herb ingredients and anticancer targets was built using Cytoscape. Furthermore, the target-pathway network was analyzed to shed light on the mechanism for the anticancer effect of TCM ingredients.

### 2.7. Evaluating the Expression Data of Hub Genes and Their Prognostic and Clinical Value

All the mRNA expression levels of hub genes expressed in HCC and in corresponding normal liver tissues were obtained using the UALCAN database [18]. The prognostic value of hub genes in HCC was further validated using Kaplan–Meier plotter database analysis [19].

The real-prognosis related hub genes acted upon by bioactive ingredients of acRoots extract in HCC were validated. The biological active ingredients and pivotal genes associated with the prognosis of HCC were identified.

The hub genes associated with HCC prognosis were obtained by combining UALCAN database and Kaplan–Meier plotter database analysis, while the real-prognosis related active ingredient of acRoots was obtained by taking the intersection of HCC prognosis-related hub genes and all biological active ingredients of acRoots.

### 2.8. Molecular Docking

Molecular docking was performed using Discovery Studio 2019. Two hub genes were included in the molecular docking simulation, including ESR1 and CAT. RCSB's protein database (PDB, https://www.RCSB.org/) was used to extract the three-dimensional crystal structures of the target proteins. We obtained the 2D conformers of active ingredients from the PubChem database (https://pubchem.ncbi.nlm.nih.gov/). The main programme employed to deal with the 3D structure of (+)-catechin was Chem3D 20.0. The 3D structure of (^+^)-catechin was prepared using the “Prepare Ligands” command to generate an effective three-dimensional conformation by Discovery Studio. The protein structures were processed by Discovery Studio, including removal of ligands and water molecules, addition of hydrogen, erasure of polyconformations, and replenishment of missing amino acid residues. Molecular docking simulation was performed by Dock Ligands (LibDock). All the obtained conformations of ligands and target proteins were analyzed to determine the interactions and binding energy of the docked conformations using Discovery Studio software.

### 2.9. Cell Culture

Human hepatoma cells (BEL-7404, SMMC-7721, Huh-7, and HepG2) and human hepatocytes (QSG-7701 and HL-7702) were cultured in an incubator at 37°C with a 5% CO_2_ atmosphere. Huh-7 and HepG2 cells were cultured in DMEM (Gibco) medium supplemented with 10% FBS (Gibco), whereas BEL-7404, SMMC-7721, QSG-7701, and HL-7702 cells were cultured in RPMI 1640 (Gibco) medium supplemented with 10% FBS (Gibco).

### 2.10. Cell Viability Assay

Human hepatoma cells SMMC-7721 and human hepatocytes HL-7702 were maintained in a medium consisting of RPMI 1640 (Gibco) medium supplemented with 10% FBS in humidified 5% CO2 at 37°C. SMMC-7721 and HL-7702 cells were plated at a density of 1,000 cells per well in 96-well plates and cultured them overnight. Then, (+)-catechin with different concentrations (0, 50,100, 150, 200, and 250 *μ*g/mL) were added to each well in a dose-dependent manner. The cells were incubated for 24, 48, and 72 h respectively. After (+)-catechin treatment, the CCK-8 working reagent (10 *μ*L/well) was added into the 96-well plates and the incubation was continued for 4 h at 37 C. Finally, optical density (OD) values was read at 450 nm using microplate reader. Cell viability was determined based on the following formula: OD value of the experimental group/OD value of the control group × 100%.

### 2.11. Treatment of Cells with Drugs

Briefly, SMMC-7721 cells were seeded in 96-well plates at 1 × 105 cells/well and treated with different concentrations of (+)-catechin (0, 50,100, 150, 200, and 250 *μ*g/mL) for 48 h at 3°C. The growth of cells was observed under a light microscope. Total RNAs were extracted from cells using 1 ml TRIzol (Sigma). A Nanodrop 100 spectrophotometer was used to determine the RNA purity and concentration. HiScript QRT SuperMix (Vazyme Biotech) was adopted to realize the reverse transcription of RNA to complementary DNA (cDNA) by resuspending 1 *μ*g of RNA in a total of 8 *μ*l RNase-free water, according to the manufacturer's instructions. Next, the expressions of ESR1 and CAT genes were assessed using quantitative real-time PCR (qRT-PCR) (Thermo Fisher Scientific).

### 2.12. Quantitative Real-Time PCR Assay

An appropriate amount of cells in each group were collected, followed by decantation of the supernatant and PBS wash. The total RNA was extracted from the cells using TRIzol solution afterward and the purity and concentration of RNA was determined using a Nanodrop100 spectrophotometry. The RNA was then reverse-transcribed to cDNA using HiScript QRT SuperMix (Vazyme biotech), which was then employed as a template to determine the mRNA expressions of ESR1 and CAT by the aid of real-time PCR. Moreover, qRT-PCR analysis was performed in a 10 *μ*L PCR reaction system, which included the following reagents: 5 *μ*L SYBR Green Mix, 0.25 *μ*L each of upstream and downstream primer sequences, 2 *μ*L template cDNA, 0.2 *μ*L Dye2, and 2.3 *μ*L RNase-free water. The thermos-cycling conditions were set as follows: (1) predenaturation at 95°C for 30 s; (2) 40 cycles of denaturation at 95°C for 5 s; and (3) amplification and extension at 60°C for 30 s. The data of the fluorescence signal was automatically detected after the steps of annealing and extension. Relative expression of each target mRNA was normalized to GAPDH mRNA expression and calculated by the 2^−ΔΔCt^ method.

### 2.13. Western Blotting

The influence of (+)-catechin on the expressions of ESR1 and CAT in protein level was assessed by Western blot. We treated SMMC-7721 cells with (^+^)-catechin of various concentrations (0, 150, 200, and 250 *μ*g/mL) for 48 h. Subsequently, the SMMC-7721 cells were collected and placed into a protein lysis buffer on ice, and the centrifugation of cells was conducted at the speed of 12,000 rpm and the temperature of 4°C for 10 min. The supernatants were preserved and used for western blot assay. BCA protein assay kit (HyClone-Pierce) was used to quantify total protein concentration of each sample, with the proteins being separated by 10% SDS‐polyacrylamide gel electrophoresis (SDS‐PAGE). Afterward, the samples were transferred to polyvinylidene difluoride (PVDF) films and were blocked at room temperature for 1 h with 5% nonfat milk, followed by overnight incubation at 4°C with primary antibody directed against ESR1 and CAT. The samples after any new round of incubation must be washed by TBST at room temperature three times (10 min for each). Thus, after the standard TBST wash, the relative secondary antibodies were incubated at room temperature for 1 h. Similarly, following another turn of TBST wash, the protein bands were visualized and detected with immobilon western chemiluminescent HRP substrate (MilliporeSigma, USA). GAPDH was utilized as an internal control.

### 2.14. Statistical Analyses

Each experiment was repeated three times independently. Comparison between groups was performed using Student's *t*-test and the Mann–Whitney *U* test. The Shapiro–Wilk test was used to check for normality of the distribution.*P*-values less than 0.05 indicated statistical significance. The statistical analyses were performed using SPSS 19.0.

## 3. Results

### 3.1. Active Ingredients of *A. chinensis* Planch Root

The various aqueous extracts of *A*. *chinensis* Planch root were composed of triterpenoids, terpenoids, polysaccharides, flavonoids, steroids, and phenolic acids. Notably, triterpenoids are considered the major active components of acRoots. 26 active ingredients were extracted from the TCMSP database in total, of which six core active ingredients satisfied the screening criteria of ADME OB ≥ 30% and DL ≥ 0.18 (Table 1).

### 3.2. Prediction and Analysis of Bioactive Ingredient Targets

**Figure 1:**
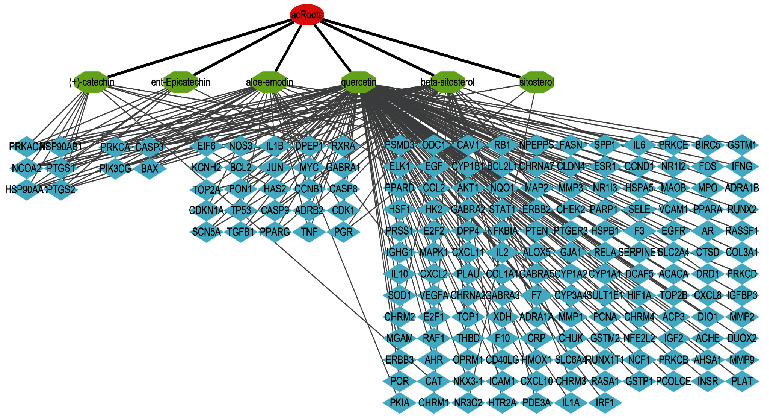
Bioactive-ingredient targets of *A. chinensis* Planch root network. The red nodes represent *A. chinensis* Planch root, the green nodes represent the bioactive ingredients (beta-sitosterol, sitosterol aloe-emodin, (+)-catechin, ent-Epicatechin, and quercetin), the blue nodes represent the main targets of action, and the edges demonstrate the relationship between the bioactive ingredients and the main targets of action.

The human disease targets of the six active ingredients were obtained from DrugBank and the UniProt public databases. According to the obtained results, 38 genes including PGR, NCOA2, and PTGS1, were selected as the candidate targets of beta-sitosterol (MOL000358). The sitosterol (MOL000359) incorporations into PGR, NCOA2, and NR3C2 were higher in human. In addition, 24 genes, including PTGS1, PTGS2, and HSP90AA1, were selected as the candidate targets of aloe-emodin (MOL000471), whereas 11 genes, including PTGS1, ESR1, and PTGS2, were selected as the candidate targets of (+)-catechin MOL000492). The ent-Epicatechin (MOL000073) incorporations into PTGS1, PTGS2, HSP90AA1, HSP90AB1, DPEP1, and PRKACA were higher in human as well. Furthermore, 148 genes including PTGS1, AR, and PPARG were selected as the candidate targets of quercetin (MOL000098). After pooling the targets and removing duplicates, a total of 175 bioactive ingredient targets of acRoots were obtained and the bioactive ingredient target network was constructed, with 181 nodes and 237 edges (Figure 1). The nodes with a degree of 1 contained 139 genes, whereas the nodes with a degree of 2 contained 25 genes (Figure 1). Moreover, the nodes with a degree of 3 contained CAPS3, PIK3CG, BAX, and PRKCA, whereas the nodes with a degree of 5 had NCOA2, HSP90AA1, HSP90AB1, PTGS1, PTGS2, and PRKACA.

### 3.3. Enrichment Analysis of the Bioactive Ingredient Targets

**Figure 2:**
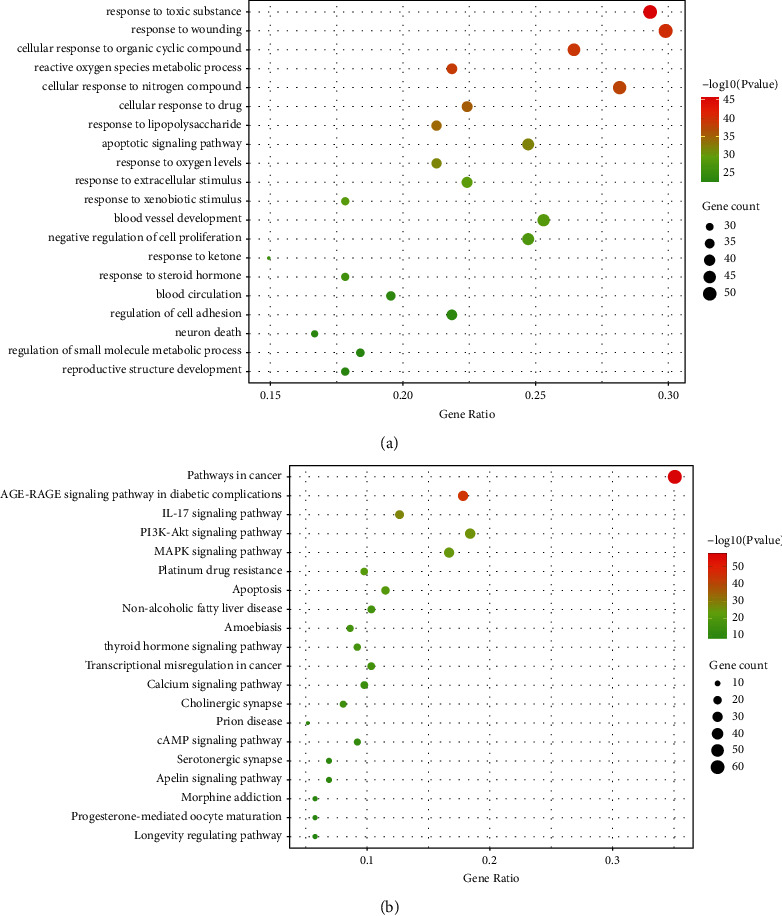
(a) The GOBP enrichment analysis bubble chart of the 175 target genes of bioactive ingredient. (b) The KEGG enrichment analysis bubble chart of the 175 target genes of bioactive ingredient.

The function of the 175 target genes of the bioactive ingredient was predicted using gene ontology (GO) biological process (BP). The GO enrichment analysis results revealed that the target genes were mainly involved in response to toxic substance, response to wounding, cellular response to organic cyclic compound, reactive oxygen species metabolic process, response to inorganic substance, cellular response to nitrogen compound, and other biological processes (Figure 2(a)). The KEGG pathways enrichment analysis showed that these genes were mainly enriched in pathways in cancer, AGE-RAGE signaling pathway in diabetic complications, IL-17 signaling pathway, PI3K-Akt signaling pathway, MAPK signaling pathway, platinum drug resistance, and other pathways (Figure 2(b)).

### 3.4. Weighted Coexpression Network Construction and Identification of Key Modules

**Figure 3:**
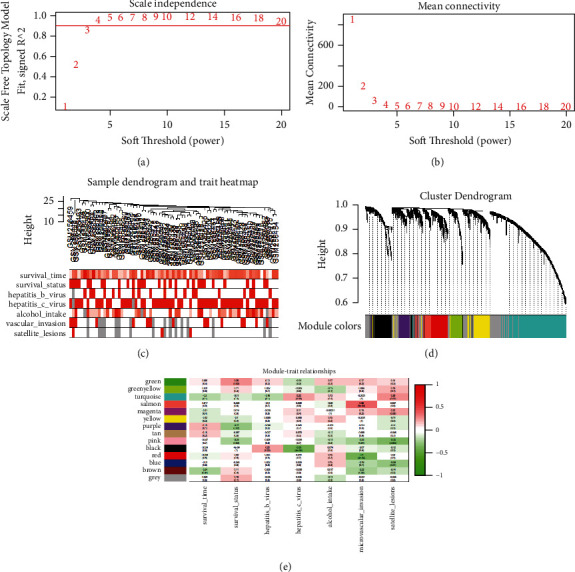
(a) Analysis of the scale-free fit index for various soft-threshold powers (*β*). (b) Analysis of the mean connectivity for various soft-threshold powers. In all, 3 was the most fit power value. (c) Sample dendrogram and trait heatmap. The clustering was in the light of the expression data of GSE10141, which contained 80 HCC samples. All genes were used for the analysis by WGCNA. The color intensity was proportional to survival time, survival status, hepatitis B virus, hepatitis C virus, alcohol intake, microvascular invasion, and satellite lesions. (d) The cluster dendrogram of genes in GSE10141. Each branch in the figure represents one gene, and each color below represents one coexpression module. (e) Heatmap of the correlation between module eigengenes and clinical information of HCC.

After a strict quality assessment and data cleaning, the expression matrices were obtained from the 80 samples in the GSE10141 training set. The clinical information, including survival time, survival status, hepatitis B virus, hepatitis C virus, alcohol intake, microvascular invasion, and satellite lesions of 80 HCC samples were obtained in coexpression analysis via WGCNA. Herein, the soft-threshold power was calculated and the power of *β* = 3 (scale free *R*^2^ = 0.9) was selected as the optimal soft-threshold to obtain a scale-free network (Figures 3(a) and 3(b)). WGCNA grouped the coexpressed genes into color-coded modules and identified 14 coexpression modules for further analysis (Figures 3(c) and 3(d)). The salmon and red modules exhibited a greater correlation with microvascular invasion than the other modules (*P* < 0.01, Figure 3(d)), the pink and blue modules were significantly associated with satellite lesions (*P* < 0.01, Figure 3(d)), and the black module was significantly negatively correlated with HCV infection (*P* < 0.01, Figure 3(d)). Finally, all genes in the above five functional modules were selected for further analysis.

### 3.5. Identification of Hub Genes Associated with Bioactive Ingredients of *A*. *chinensis* Planch Root Extract

**Figure 4:**
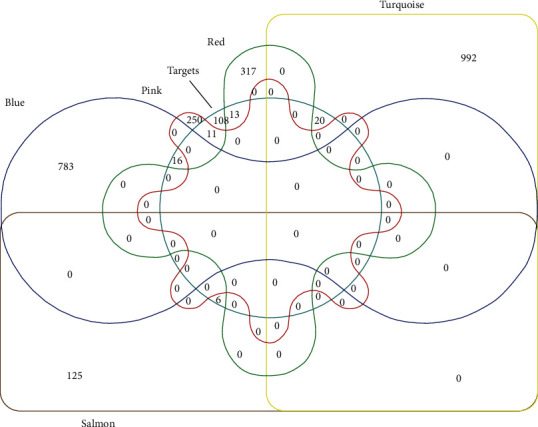
The Venn diagram of the targets selected from both modules-related genes and bioactive-ingredient targets of *A. chinensis* Planch root.

**Figure 5:**
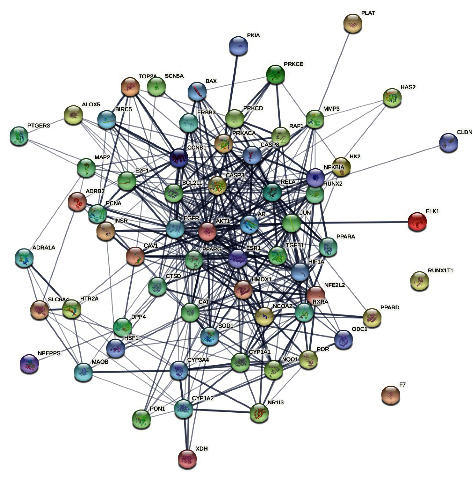
The PPI network of the 66 potential anti-HCC bioactive-ingredient targets.

Following the coexpression of the acRoots active ingredient target genes with the selected modules-related genes, a total of 66 antitumor targets were identified. Among them, results showed that the turquoise module had 20 antitumor target genes, such as NFE2L2, ESR1, TGFB1, MMP3, and so on. The blue module had 16 antitumor target genes, including CASP8, PRKCD, HAS2, RUNX1T1, and PPARD, whereas the pink module had 11 antitumor target genes, including RXRA, RAF1, PCNA, PRKCE, and ERBB3. The red module had 13 antitumor target genes, including XDH, PON1, F7, CYP1A1, and NR1I3, whereas the salmon module had six antitumor target genes, such as CCNB1, BIRC5, E2F1, ADRA1A, TOP2A, and HIF1A (Figure 4). Consequently, a visual PPI network of the 66 potential antitumor targets was constructed containing 66 nodes and 378 edges in total (Figure 5). Notably, analysis of the topological characteristics of the obtained PPI was based on the major parameter of “degree ≥ 20.” The 11 key target genes we mapped in this network include AKT1 (degree = 40), Casp3 (degree = 33), EGFR (degree = 32), JUN (degree = 32), ESR1 (degree = 30), CAT (degree = 28), PPARG (degree = 23), AR (degree = 23), HIF1A (degree = 21), RELA (degree = 20), and BCL2L1 (degree = 20).

### 3.6. The Hub Genes of GO and KEGG Pathway Enrichment Analyses

**Figure 6:**
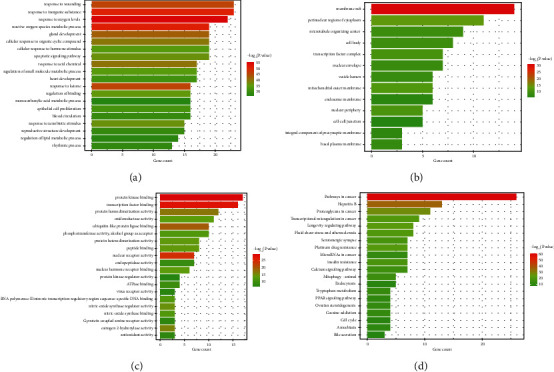
(a) The distribution of GO enrichment items in the biological processes (BP) of 66 antitumor target genes of acRoots. (b) The distribution of GO enrichment items in the Cellular Component (CC) of 66 antitumor target genes of acRoots. (c) The distribution of GO enrichment items in the molecular function (MF) of 66 antitumor target genes of acRoots. (d) The distribution of KEGG enrichment items of 66 antitumor target genes of acRoots.

The 66 antitumor target genes of acRoots were categorized into three functional groups (BP: biological process, CC: cellular component, and MF: molecular function). The genes in the BP group were mainly enriched in response to wounding, response to inorganic substance, and other processes (Figure 6(a)); the genes in the CC group were mainly enriched in membrane raft, perinuclear region of cytoplasm, and other processes (Figure 6(b)); and the genes in the MF group were significantly enriched in protein kinase binding, transcription factor binding, and other binding processes (Figure 6(c)). The KEGG pathway analysis results showed that these genes were mainly involved in pathways in cancer (Figure 6(d)).

### 3.7. Validation and Prognosis Value of Key Genes

**Figure 7:**
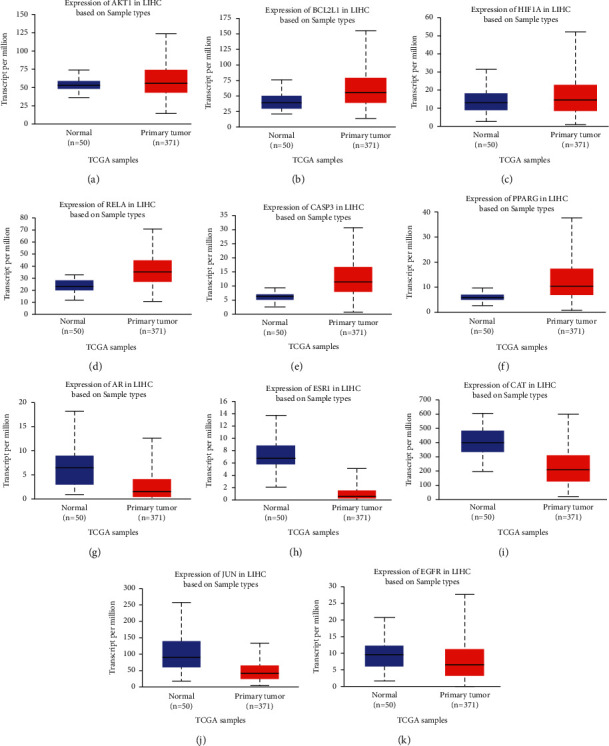
Effect of expression of the 11 hub genes (AKT1, BCL2L1, HIF1A, RELA, Casp3, PPARG, CAT, JUN, ESR1, AR, and EGFR) closely associated with acRoots by TCGA HCC data in UALCAN.

To further validate the clinical significance of the 11 hub genes, the expression levels of the hub genes in human normal liver and HCC tissues obtained from TCGA database were analyzed on the ground of data mining via UALCAN. The results showed that AKT1, BCL2L1, HIF1A, RELA, CASP3, and PPARG were highly expressed in HCC tissues compared to normal tissues (Figure 7). Conversely, the expression levels of AR, ESR1, CAT, and JUN were significantly downregulated in the HCC tissues compared to the adjacent normal liver tissues. It is noteworthy that there was no significant difference in the expression of EGFR.

**Figure 8:**
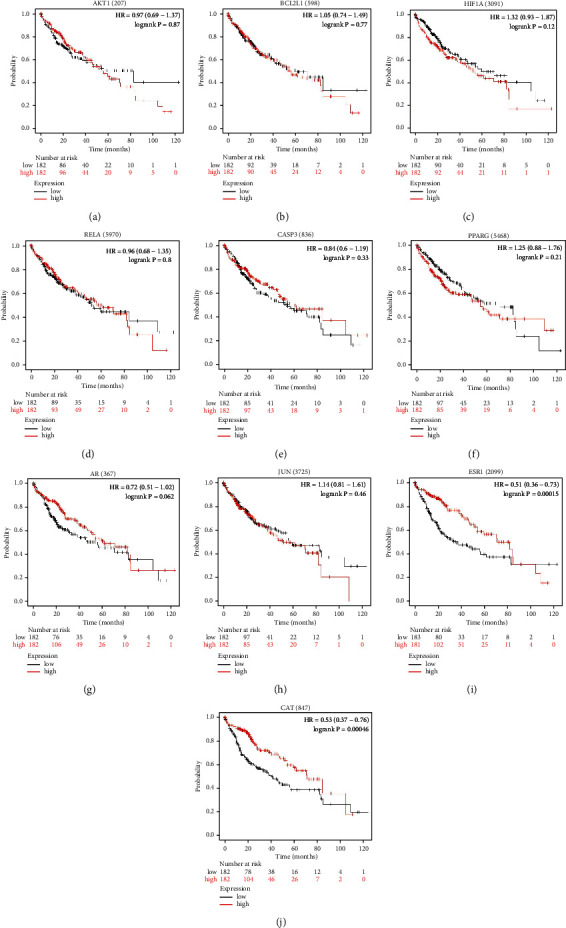
The results of overall survival analysis of the 10 hub genes (AKT1, BCL2L1, HIF1A, RELA, Casp3, PPARG, AR, JUN, ESR1, and CAT) closely associated with acRoots using Kaplan–Meier plotter database.

**Figure 9:**
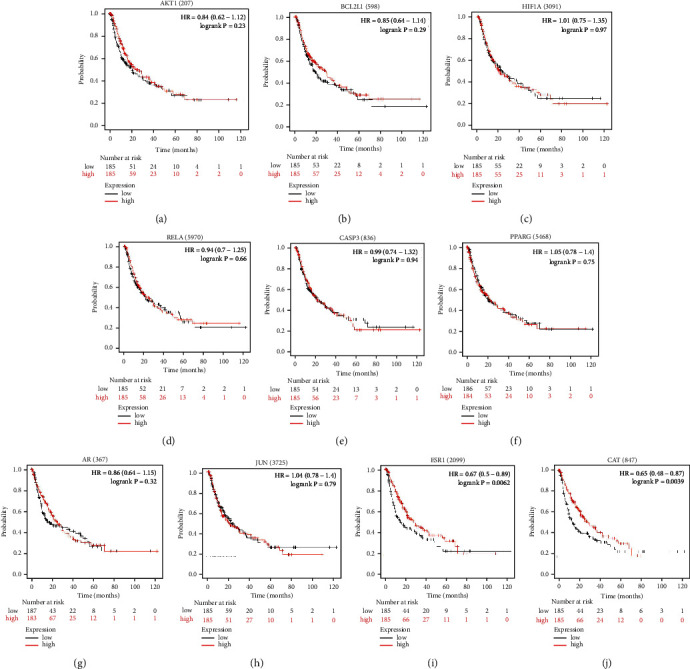
The results of progression-free survival analysis of the 10 hub genes (AKT1, BCL2L1, HIF1A, RELA, Casp3, PPARG, AR, JUN, ESR1, and CAT) closely associated with acRoots using Kaplan–Meier plotter database.

The correlation between expressions of the 11 hub genes and the overall survival (OS) of patients was investigated by analyzing Kaplan–Meier plotter database. The results demonstrated that the overall survival rate of patients exhibiting significantly high ESR1 and CAT expression was greatly increased compared to the survival rate of patients demonstrating low ESR1 and CAT expression (*P* < 0.01) (Figure 8). However, the expressions of AKT1, CASP3, AR, JUN, PPARG, HIF1A, RELA, and BCL2L1 did not have a distinct effect on OS (*P* > 0.05) (Figure 8). Further analyses illustrated that ESR1 and CAT were overexpressed in patients experiencing longer progression-free survival (PFS) (*P* < 0.01) (Figure 9). Moreover, it is evident that there was a certain correlation between the expression of hub genes and prognostic values on patient survival in the HCC patient group.

### 3.8. Molecular Docking

**Figure 10:**
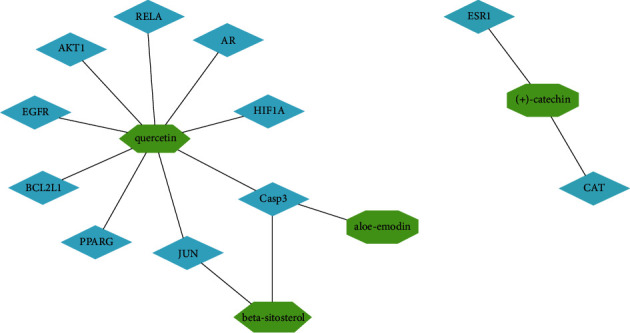
Molecular docking patterns of (+)-catechin with targets. (A) ESR1 protein-(+)-catechin, (B) CAT protein-(+)-catechin.

Molecular docking studies were carried out to identify the interactions between the active ingredient ((+)-catechin) and HCC-related potential target genes (ESR1 and CAT) at the molecular level. We calculated the precision of docking between (^+^)-catechin and target proteins, with the LibDock score of 71.1138 and 115.911. The higher LibDock score implies better bonding. The results unveil that (+)-catechin has a good binding ability to both targets (Table 2). 4 hydrogen bonds between (+)-catechin and 4 residues (HIS-516, HIS-524, HIS-547, and LYS-520) firmly connect (+)-catechin with 1ERE (ESR1), while another 4 hydrogen bonds between (+)-catechin and other residues (glu-71 and HIS-175) form stable binding between (+)-catechin and 1DGF (CAT). Furthermore, other binding forces including carbon hydrogen bond, pi-cation, pi-sulfur, and pi-alkyl bonds were also found in the (+)-catechin- ESR1 binding, whereas forces such as van unfavorable donor-donor, pi-anion, and pi-alkyl bonds were proven to be existed in the (^+^)-catechin-CAT binding as well. More information was detailed in Figure 10.

### 3.9. Impact of (+)-Catechin on the Cell Viability of SMMC-7721 Cells and HL-7702

**Figure 11:**
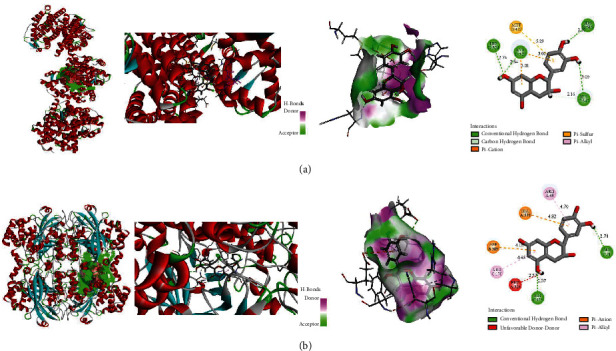
(a) SMMC-7721 cell viability was evaluated via CCK-8 assay. The SMMC-7721 cells were treated with (+)-catechin of different concentrations (0, 50, 100,150, 200, and 250 *μ*g/mL) in a dose-dependent manner for 24, 48, and 72 h. (b) HL-7702 cell viability was evaluated via CCK-8 assay. The HL-7702 cells were treated with (+)-catechin of different concentrations (0, 50, 100,150, 200, and 250 *μ*g/mL) in a dose-dependent manner for 24, 48, and 72 h. (c) Changes in mRNA expression of ESR1 in SMMC-7721 liver cancer cells when treated with different concentrations of (+)-catechin. (d) Changes in mRNA expression of CAT in SMMC-7721 liver cancer cells when treated with different concentrations of (+)-catechin. (e) Influence of (+)-catechin on the ESR1 and CAT expression in SMMC-7721 cells investigated by Western blot assay. The statistical significance was presented at *P* < 0.05 (^*∗*^), *P* < 0.01 (^*∗∗*^), or *P* < 0.001 (^*∗∗∗*^).

To investigate the role of (+)-catechin in human hepatocarcinoma, we first examined the cytotoxic effect of (+)-catechin on SMMC-7721 cells. As shown in Figure 11(a), the cell viability of SMMC-7721 cells treated with 50, 100, 150, 200, and 250 *μ*g/ml (+)-catechin for 24 h remained unchanged. When we prolonged the incubation time to 48 h or 72 h, (+)-catechin with concentrations of 100, 150, 200, and 250 *μ*g/ml remarkably reduced the cell viability of SMMC-7721 cells compared with those low-concentration (+)-catechin (0 and 50 *μ*g/ml). Hence, it can be argued that the cell viability of SMMC-7721 cells decreases dose-dependently and time-dependently by (+)-catechin treatment. However, the study revealed that the cytotoxic effect of the (+)-catechin with concentrations of 50, 100, 150, 200, and 250 *μ*g/ml on HL-7702 was not significant for 24 h, 48 h, or 72 h.

### 3.10. Effect of (+)-Catechin on ESR1 and CAT mRNA Expression Levels in Human Hepatocarcinoma Cells

**Figure 12:**
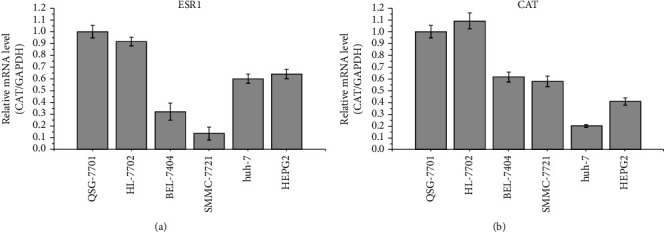
The network based on the 11 hub genes and four bioactive ingredients of *A. chinensis* Planch root. The green nodes represent the bioactive ingredients (beta-sitosterol, aloe-emodin, (+)-catechin, and quercetin), the blue nodes represent the hub genes, and the edges represent the relationship between the bioactive ingredients and the 11 hub genes.

**Figure 13:**
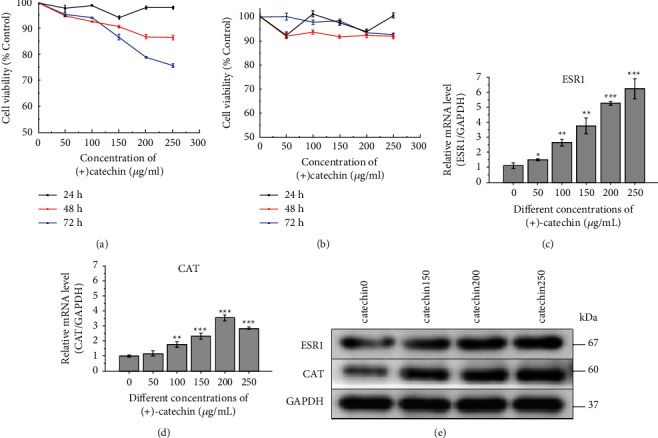
Comparison of the expression levels of ESR1 (a) and CAT (b) among BEL-7404, SMMC-7721, Huh-7, and HEPG2 liver cancer cells and QSG-7701, HL-7702 normal liver cells.

The mRNA expression levels of ESR1 and CAT associated with the prognosis of HCC were validated using database analysis. We further analyzed the effects of four anti-HCC bioactive ingredients of acRoots, including quercetin, (+)-catechin, beta-sitosterol, and aloe-emodin, and found that (+)-catechin could affect the HCC prognosis-related genes ESR1 and CAT(Figure 12). The in vitro experiment further confirmed that ESR1 and CAT were downregulated in HCC cell lines compared to normal liver cells (Figure 13). The outcomes also suggested that (+)-catechin could gradually heighten the ESR1 and CAT expression levels due to its correspondingly increased concentration (Figures 11(b) and 11(c)).

### 3.11. Effect of (+)-Catechin on the Expression of Prognosis-Related HCC

ESR1 and CAT are critical molecules associated with the prognosis of HCC. The Western blot assay was utilized to examine the changes of prognosis-related proteins ESR1 and CAT after the 48-hour treatment of SMMC-772 cells with (+)-catechin at different concentrations (0, 150, 200, and 250 *μ*g/mL). As the concentration of (+)-catechin increased, the expression of the protein ESR1 and CAT increased significantly (Figure 11(d)).

## 4. Discussion

There is accumulated evidence that acRoots has a wide range of pharmacological activities, including anticancer, antioxidant, anti-inflammatory, and immunoregulatory [6]. Although some empirically underscore acRoots' potential antitumor effect on HCC, the underlying mechanisms of such an effect remain understudied [20, 21]. The occurrence and development of HCC is a complex process involving multiple targets and pathways [1, 3]. Since genes and the related pathways are difficult to identify one by one, we comprehensively investigated acRoots' potential targets in HCC and subsequently its mechanisms of action by means of the network pharmacology method.

According to the aforementioned results, 6 active ingredients in acRoots, including beta-sitosterol, sitosterol, aloe-emodin, (+)-catechin, ent-Epicatechin, and quercetin, were identified. Among these ingredients, beta-sitosterol (BS) reportedly has antitumor activities against breast cancer, prostate cancer, colon cancer, stomach cancer, and liver cancer [22]. It has also been shown to regulate multiple cell signaling pathways, such as cell cycle, apoptosis, proliferation, survival, invasion, angiogenesis, metastasis, and inflammation [22–24]. Further, one corroborated that beta-sitosterol could promote ROS production, activate AMPK, acetyl-CoA carboxylase (ACC), and JNK, and attenuate the phosphorylation of AKT and the expression of cyclooxygenase-2 and VEGF [23]. In a similar vein, Vo et al. [22] found that beta-sitosterol exerts an anticancer effect by activating caspase-3- and caspase-9-mediated apoptotic signaling in HCC cells. From a holistic perspective, these mechanisms suggest that triterpenoids are able to exert an antitumor effect. Moreover, one paper probing the antitumor activity on multiple types of cancer cell lines (T24 cells, SW480 carcinoma cells, AGS cells, and Huh-7 cells) upheld that aloe-emodin could induce cell cycle arrest and apoptosis, modulate immune signals, and alter cell mobility [25]. A later study, focusing on oral squamous cell carcinoma, indicated that treatment of cancer cells with aloe-emodin significantly upregulated the expression of caspase-9 and caspase-3 and induced apoptosis [26]. When it comes to (+)-catechin, many reported that its anticancer effects were intimately correlated to the contents of antioxidants and enzyme inhibitors, making it capable of scavenging oxygen radicals [27, 28]. The treatment of cancer cells with (+)-catechin resulted in a significant decrease in catalase level and an upgrade of the ROS level, which hence led to the inhibition of tumor cell growth and ultimately pushed the tumor toward apoptosis [29]. Similarly, another paper concerned with liver cancer cells (HepG2 cells) found that (+)-catechin downregulated Bcl-2 and upregulated Bax, caspase-3, caspase-9, and p53, thereby inducing apoptosis and inhibiting G₂/M phase in cell cycle analysis [27]. Ent-Epicatechin has proven to have a protective effect on healthy tissue cells, and it can also interfere with the processes of cancer signaling, metabolism, and proliferation [30, 31]. In addition, ent-Epicatechin stimulates mitochondrial oxidative phosphorylation through Erk and/or EGFR signaling pathways, thereby interfering with Warburg metabolism and inhibiting NF-*κ*B, Akt, and histone acetyltransferases (HATs) [30]. It is also evident that ent-Epicatechin treatment can sensitize cancer cells to radiation treatment or chemotherapy [31], as cancer cells are more susceptible to apoptosis. Quercetin reportedly exerts extensive biological effects, among which the most prominent effects are anticancer and cancer preventative effects in many types of malignancies [32]. Quercetin also reduced stabilization of *β*-catenin and HIF-1*α*, induced activation of caspase-3, and inhibited Akt, mTOR, and ERK phosphorylation in cancers, denoting that quercetin facilitates the loss of cell viability, apoptosis, and autophagy through modulation of PI3K/Akt/mTOR, Wnt/*β*-catenin, and MAPK/ERK1/2 pathways [33, 34]. More recently, Hisaka et al. [32] asserted that quercetin had antiproliferative and apoptotic effects on 13 liver cancer cell lines, suggesting that quercetin suppressed HCC proliferation and induces apoptosis. Ren et al. [33] yielded a similar finding that quercetin accelerated the cleavage of caspase (caspase-9 and caspase-3), which induced apoptosis, and impeded PI3K/Akt and ERK signaling pathways in regulation of antitumor activity in liver cancer cells. Overall, the studies described above collectively shed light on the potential performance-enhancing effects of the 6 active ingredients identified in acRoots, which can be capitalized on to improve the therapeutic outcome for cancer patients.

In spite of the active ingredients in acRoots for potential anticancer activity, this study further identified 11 key target genes, namely, AKT1, Casp3, EGFR, JUN, ESR1, Cat, PPARG, AR, HIF1A, RELA, and BCL2L1, by combining network pharmacology with WGCNA analysis. Notably, our results are consistent with findings reported in previous studies [35–44]. Furthermore, GO and KEGG pathway analyses were performed to further illustrate the potential anticancer biological functions and regulatory pathway of acRoots. The results implied that acRoots exerted its antitumor effects on HCC cells mainly through inhibiting proliferation and angiogenesis and promoting apoptosis. KEGG annotation and pathway enrichment analysis indicated that the major targeted pathways of acRoots were PI3K-AKT pathway, JAK-STAT signaling pathway, PPAR signaling pathway, estrogen signaling pathway, HIF-1signaling pathway, androgen receptor signaling pathway, and apoptosis signaling pathway (Figure 9). Although such a finding is endorsed by a recent scholarly endeavour arguing that acRoots have a good capacity to regulate the proliferation, cycle stagnation, and apoptosis of HCC cells through the p-Akt/PTEN pathway [45], there remains a scarcity of studies probing alternative molecular mechanisms of acRoots. Consequently, future studies are encouraged to assess the generalizability of our findings.

Finally, we performed a comprehensive bioinformatics analysis to explore the expression of the 11 hub genes in HCC as well as their prognostic significances. The findings indicate that ESR1 and CAT were closely associated with overall survival and longer progression-free survival of HCC patients. Previous studies found that ESR1 functions as a tumor suppressor gene in HCC and mediates apoptosis of liver cancer cells [40, 46]. Also, the expression of ESR1 was found to be positively related with the prognosis in HCC (i.e., the greater the expression of ESR1 is, the better the HCC prognosis is). The CAT gene encodes catalase, which induces the decomposition of the H2O2 generated by cancer cells to produce oxygen, thereby attenuating the hypoxia condition in the tumor microenvironment [41]. The results obtained in this study also indicated that treatment with (+)-catechin could significantly increase the expression levels of ESR1 and CAT in human hepatocellular carcinoma cells. In a nutshell, our findings confirmed that acRoots could act on HCC by regulating the aforementioned target genes, thereby improving the prognosis of HCC patients.

## 5. Conclusion

To conclude, the present study employed a systems network pharmacology approach to identify 11 effective core targets of bioactive ingredient in acRoots. The 4 bioactive ingredients, including quercetin, (+)-catechin, beta-sitosterol, and aloe-emodin, were found effective in mitigating HCC. Nevertheless, further results indicated that only ESR1 and CAT, among the 11 core genes, were closely associated with the prognosis of HCC patients. This study predicted several signaling pathways through which acRoots could participate in the treatment of HCC. Also, ESR1 and CAT presumably induced the related pathways, such as the estrogen signaling pathway and the longevity regulation pathway. Further, (+)-catechin was identified as the only bioactive ingredient of acRoots that acted on both ESR1 and CAT, and hence it was thought to play a crucial role in improving the prognosis of HCC patients. Finally, this study contributes to the body of knowledge by providing a conceptual guidance for the clinical treatment of HCC and a methodological design potentially applicable in the future comprehensive analysis of the underlying mechanisms of TCMs.

## Figures and Tables

**Table 1:** Information on screened active ingredients of Actinidia chinensis Planch root.

Id	Active ingredients	OB (%)	DL
MOL000358	Beta-sitosterol	36.91	0.75
MOL000359	Sitosterol	36.91	0.75
MOL000471	Aloe-emodin	83.38	0.24
MOL000492	(+)-catechin	54.83	0.24
MOL000073	Ent-epicatechin	48.96	0.24
MOL000098	Quercetin	46.43	0.28

acRoots, Actinidia chinensis Planch root extract.

**Table 2:** Docking simulation for active molecules and targets of HCC.

Molecular name	Targets	Pbd id	Residue involved in H bonding	LibDock score
(+)-catechin	ESR1	1ERE	HIS-524, HIS-547, HIS-516, LYS-520	71.1138
(+)-catechin	CAT	1DGF	GLU-71, HIS-175	115.911

## Data Availability

The data used to support the findings of this study are included within the supplementary information file.

## Supplementary Materials

Supplementary Materials
*Supplemental File (1)*. The graphical abstract. *Supplemental File (2).* Active ingredients of *Actinidia chinensis* Planch. root. *Supplemental File (3)*. GSE10141_sample. *Supplemental File (4)*. GSE10141_sample_expression. *Supplemental Files (5–10).* Cell experimental study data.
