# Supplementary material for: Systems Network Pharmacology-Based Prediction and Analysis of Potential Targets and Pharmacological Mechanism of Actinidia chinensis Planch. Root Extract for Application in Hepatocellular Carcinoma
Source: Evid Based Complement Alternat Med. 2022 Sep 20;2022:2116006. doi: 10.1155/2022/2116006 (PMC9526650; doi:10.1155/2022/2116006)
Supplement: Supplementary Materials — Supplemental File (1). The graphical abstract. Supplemental File (2). Active ingredients of Actinidia chinensis Planch. root. Supplemental File (3). GSE10141_sample. Supplemental File (4). GSE10141_sample_expression. Supplemental Files (5–10). Cell experimental study data. [file 2116006.f1.zip › supplementary file(1) (1).doc]

Actinidia chinensis Planch root

OB+DL	screening

Bioactive ingredients

Targets	fishing

screening

bioactive ingredient-targets network

beta-sitosterol

sitosterol

aloe-emodin

(+)-catechin

ent-Epicatechin

quercetin

GO and KEGG Enrichment Analysis

bioactive ingredient-targets

Hepatocellular carcinoma

WGCNA

key modules genes

hub genes

GO and KEGG Enrichment Analysis

PPI

Network

hub genes

expressions

hub genes

prognostic value

HCC prognosis-related hub genes and biological active ingredient

Cell experiments

Molecular docking
