# Supplementary material for: Systems Network Pharmacology-Based Prediction and Analysis of Potential Targets and Pharmacological Mechanism of Actinidia chinensis Planch. Root Extract for Application in Hepatocellular Carcinoma
Source: Evid Based Complement Alternat Med. 2022 Sep 20;2022:2116006. doi: 10.1155/2022/2116006 (PMC9526650; doi:10.1155/2022/2116006)
Supplement: Supplementary Materials — Supplemental File (1). The graphical abstract. Supplemental File (2). Active ingredients of Actinidia chinensis Planch. root. Supplemental File (3). GSE10141_sample. Supplemental File (4). GSE10141_sample_expression. Supplemental Files (5–10). Cell experimental study data. [file 2116006.f1.zip › supplementary file(9).doc]

CCK8
